# Patient Assessment of Care with Chronic Cardiovascular Disorders and Its Relationship with Self-Efficacy: A Cross-Sectional Study

**DOI:** 10.3390/healthcare11152189

**Published:** 2023-08-02

**Authors:** Aidah Sanad Alqarni, Eddieson Pasay-An, Awad Eid Alshammari, Ferdinand Gonzales, Lorraine Estadilla, Kawther Eltayeb Ahmed, Lizy Sonia Benjamin, Andrew Ngo, Hanan Awad Moawad Elmashad, Dawlat Ahmed mahmoud Gharib, Salman Amish Alshammari

**Affiliations:** 1 Medical Surgical Nursing Department, College of Nursing, King Khalid University, Abha 62521, Saudi Arabia; 2 College of Nursing, University of Hail, Hail City 2440, Saudi Arabia; 3 Nursing Department, Ministry of Health, Hail City 55421, Saudi Arabia; 4 Department of Community, Psychiatric and Mental Health Nursing, College of Nursing, Qassim University, Buraydah 51452, Saudi Arabia; 5 Maternity and Neonatal Nursing Department, King Khalid University, Abha 62521, Saudi Arabia

**Keywords:** assessment of care, chronic cardiovascular disease, correlational, patient, self-efficacy

## Abstract

Introduction: Patients’ assessment of care navigating cardiovascular disorders is imperative in improving the quality of care provided. The purpose of this study was to explore the perspectives of people living with cardiovascular disorders on the care they received and its relationship with general self-efficacy. Methods: This investigation employed a cross-sectional correlational approach. The study sample was comprised of patients with cardiovascular disorders from both the King Khalid Hospital and the King Salman Specialist Hospital, in Hail City, Saudi Arabia. Convenience sampling was used, resulting in 104 participants. A survey using a self-administered questionnaire was employed to collect the data, which ran from 10 March to 20 May 2023. Results: The participants perceived that they occasionally (2.75 ± 1.053) received care, and they perceived themselves to have better self-efficacy (25.28/40). Of note, the age (0.062), years of being diagnosed with having the disease (−0.174), sex (0.180), educational attainment (0.125), and occupation (0.206) were found to have no significant relationship with the patient assessment of care with chronic cardiovascular disease (PACIC). However, civil status (0.867) was found to have a strong positive correlation to the PACIC. No significant relationship was found between age and GSE (0.070) and PACIC (0.62), civil status with GSE (0.013), years being diagnosed with having the disease with GSE (0.095), and PACIC (0.174) educational attainment with GSE (0.088) and PACIC (0.125) or occupation (0.115) with GSE. However, sex (0.795) was found to have a strong correlation with general self-efficacy (GSE). Of note, patient activation (0.390) and goal setting (0.360) had a moderate positive correlation while problem solving (0.228) and follow-up (0.278) had a weak positive correlation to GSE. Meanwhile, the delivery system (0.507) had a strong positive correlation to GSE. This study illuminates the value of self-efficacy and patient involvement as self-management techniques for cardiovascular illnesses. Future cardiovascular illness self-management initiatives should concentrate on enhancing patient self-efficacy by adopting the PACIC.

## 1. Introduction

The term ‘cardiovascular disease’ (CVD) refers to a group of disorders, including coronary artery diseases (e.g., angina, myocardial infarction), that affect the heart and blood vessels [1]. Roughly 18 million people die from CVD, and approximately 35 million have nonfatal cardiovascular events [2]. Nonetheless, up to 90% of cardiovascular disease is thought to be preventable with good food, exercise, and limiting alcohol consumption, with the avoidance of risk factors such as tobacco smoke [3]. To this end, patients with CVD need to control their illnesses and avoid complications, and they must learn how to control their own care. In perspective, the patient’s assessment of the care for navigating cardiovascular disorders is imperative to improving the quality of the care provided.

CVD is a class of diseases that involves the heart or blood vessels [1]. It includes coronary artery diseases (CAD), such as angina and myocardial infarction, which is commonly known as a heart attack. Other CVDs include stroke, heart failure, hypertensive heart disease, rheumatic heart disease, cardiomyopathy, abnormal heart rhythms, congenital heart disease, valvular heart disease, carditis, aortic aneurysms, peripheral artery disease, thromboembolic disease, and venous thrombosis [1]. While patients diagnosed with cardiovascular diseases need medical attention (e.g., medication, psychological intervention, lifestyle changes) from a variety of providers and professions [4], validation of the quality of the care provided is also important. This ensures setting up a quality control system, developing and executing quality indicators, and ensuring continuous improvement [5], which necessitates a unique and multidimensional approach that can eventually advance the self-efficacy of the patients.

Understanding the progression and management of CVD, including how people make lifestyle changes to compensate for it, requires a psychological understanding of aspects such as self-efficacy [6]. Improving self-efficacy can assist patients with chronic diseases in optimizing their health and well-being outcomes [7]. To accomplish this, the patients themselves need to evaluate the care provided by the healthcare personnel based on quality measures that could improve their confidence or self-efficacy. Willis [8] maintained that a person’s self-efficacy has a direct impact on behaviour change, while encouragement or discouragement from one’s social support system has an impact. Individuals with chronic illnesses must learn to manage their own care to better control and avoid further complications of their illnesses [9]. To accomplish this requires improvements and strategies by way of a planned and proactive approach to care and long-term planning, which necessitates an ongoing collaborative engagement between patients and healthcare practitioners [10].

Patients’ self-efficacy is thought to be significant in managing life with CVD and the potential for creating and adhering to a rehabilitation plan. The role of patients in managing their care is very important where they must take an active part in the delivery of health care and the services that go along with it and these services must be adapted to the requirements and expectations of the patients [11]. For example, through healthy behaviour and illness management, people and their families can maintain their own well-being [12]. People who practice self-care maintenance stick to the routines required to preserve their mental and physical well-being. Self-care monitoring involves paying attention to one’s own body and listening for changes in signs and symptoms. People respond to signs and symptoms by managing their own treatment when they appear [12].

The well-known chronic care model (CCM) [13] offers a practical paradigm for improving evidence-based chronic care [14]. The purpose of CCM-based healthcare is to produce an informed, engaged patient who interacts with a proactive, prepared practice team, resulting in productive encounters and better outcomes [15], such as patient self-efficacy [13]. Reviews from scholars have shown that using a CCM to carry out interventions enhances performance, health outcomes [13], decision making, and irrefutable information systems [15]. However, to the best of the knowledge of the current researchers, the perspectives of people living with cardiovascular diseases are scarce and thus need to be represented in the literature.

Assessment of chronic cardiovascular care with the implementation of CCM among patients with cardiovascular illnesses and its relationship to self-efficacy is crucial since it forms the cornerstone of bettering the standards of treatment. In addition, it also helps the patient to improve his or her own health outcomes and quality of life. It is in this context that a planned and proactive approach to care is of significance in leading the patient to manage his or her health with self-efficacy. We hypothesize therefore that PACIC domains correlate with GSE. This study aimed to explore the perspectives of people living with cardiovascular disorders on the care received and its relationship with general self-efficacy.

## 2. Methods

### 2.1. Design

The researchers employed a cross-sectional-correlational approach to explore the perspectives of the people living with cardiovascular disorders on the care they received and its relationship with general self-efficacy.

### 2.2. Setting/Sampling

The study took place at both the King Khalid Hospital and the King Salman Specialist Hospital, Hail City where they cater for patients with cardiac problems. There were 104 participants who were invited to participate in the study. The Raosoft sample size calculator (http://www.raosoft.com/samplesize.html, accessed on 3 January 2023) was used to identify the number of needed participants for this study. Based on the 142 overall population of patients with CVD in the two hospitals, the required number of participants was 104 patients yielded from convenience sampling. The participants were people who had been convalescing from cardiovascular disorder. Inclusion criteria were (a) those who had recuperated from cardiac disorders (e.g., myocardial infarction, heart failure), (b) were in good health condition during the time of the study, (c) were willing to take part in the study, and (d) with no comorbidities such as diabetes mellitus, depression, etc. They were excluded if (a) they had been diagnosed only a week before the study, (b) or they were admitted to the hospital for any scheduled procedure.

### 2.3. Data Collection

Data collection started after obtaining the approval of the hospital directors of each participating hospital. An orientation was conducted for the invited participants. Its purpose was to explain the aim of the study, the extent of participation, and their rights. A paper-based questionnaire form was given to the participants. It included instructions to read the informed consent before proceeding to answer the questionnaire. To ensure that the health condition of the participants was not compromised, the researchers used a paper-pencil-based questionnaire with the supervision of the researchers and the nurse on duty. A minimum of 10 min was given for the participants to answer the questionnaire, and they were able to extend that time based on their individual pacing. This study was conducted between February and March 2023.

### 2.4. Questionnaire

The researchers adopted the Arabic-translated PACIC by Alharbi and colleagues [16]. The PACIC is a 20-item self-report questionnaire that evaluates the CCM implementation from the patient’s point of view. On a 5-point scale, ranging from 1 (never) to 5 (always), patients are asked to rate the frequency with which they receive such care. The overall scores could be calculated for the PACIC (items 1–20) [17]. Accordingly, the Arabic-translated questionnaire displayed acceptable psychometric quality. The Cronbach’s alpha was greater than 0.9, while the inter-item correlation varied between 0.36 and 0.56. Exploratory factor analysis found a single-factor structure.

The second questionnaire was the self-efficacy scale developed by Schwarzer and Jerusalem [18]. That scale ranges from 1 (not at all true), to 2 (hardly true), 3 (moderately true), and 4 (exactly true), with the total score ranging from 10 to 40, and the higher values indicating more self-efficacy. The researchers translated the GSE instrument into Arabic, a language that is commonly spoken in the region. Two panels of experts with doctorates in language communication carried out the forward and reverse translations. The questionnaires were validated by a group of experts with substantial experience of cardiovascular illnesses, considerable experience in psychometric testing, and experience as research consultants. Each question was examined during the first round of validity testing to determine how well it matched the survey’s objectives and how clearly it was phrased. To increase the relevance of the content, the first round suggestions were included in the second round. After the face validity, content validity testing, in particular S-CVI/UA, was conducted. The survey’s SCVI/UA score of 0.874 indicated that all the items were agreed upon.

### 2.5. Data Analysis

The Statistical Package for the Social Sciences (SPSS) Version 21 was used to analyse the data. Frequency and percentage were utilised to determine the demographic profile of the respondents. The normality test was employed to determine the distribution of the data under the presumption that they were normally distributed. The Kolmogorov–Smirnov test result was higher (0.91; *p* = 0.05), indicating that the data were normally distributed. Therefore, the correlation (bivariate *r*) was used to test whether a statistically significant relationship existed between the variables.

## 3. Results

The demographic characteristics of the participants are presented in Table 1. Of the 104 participants, 62.5% were over 30 years old, and 66.3% were married. Half of the participants (50%) were diagnosed to have had cardiovascular disease for three or more years. The participants were dominated by females (58.7%). Over half of them had attained high school level (51.9%), and nearly 60% were employed.

The correlations between the demographics of the GSE and the PACIC are presented in Table 2. Of note, the age (0.062), years of being diagnosed with the disease (−0.174), sex (0.180), educational attainment (0.125), and occupation (0.206) were found to have no significant relationship with the PACIC. However, the civil status (0.867) was found to have a positive strong correlation with it. Further, no significant relationship was found between age and GSE (0.070) and PACIC (0.62), civil status with GSE (0.013), years being diagnosed with the disease with GSE (0.095), and PACIC (0.174) educational attainment with GSE (0.088) and PACIC (0.125) or occupation (0.115) with GS. However, sex (0.795) was found to have a strong correlation with the GSE. Finally, there was a strong correlation between the GSE and the PACIC (0.881).

The levels of the PACIC and the GSE as perceived by the patients are presented in Table 3. The overall perceived PACIC was 2.75 ± 1.053 ranging from 2.58 to 3.07. Further, participants perceived themselves to have better self-efficacy (25.28/40). Of note, patient activation (0.390) and goal setting (0.360) had a moderate positive correlation while problem-solving (0.228) and follow-up (0.278) had a weak positive correlation to GSE. Meanwhile, the delivery system (0.507) had a strong positive correlation to GSE.

## 4. Discussion

### 4.1. Factors Improving Self-Efficacy

This study aimed to explore the perspectives of people living with cardiovascular disorders on the care they received and its relationship with general self-efficacy. Of note, the age, years of being diagnosed with the disease, sex, educational attainment, and occupation were found to have no significant relationship with the PACIC. These demographic factors imply that the PACIC scores remain constant regardless of age, the number of years since a disease diagnosis, sex, level of education, and occupation. In contrast to the results of this study, studies by both Rosemann et al. [19] and Krucien et al. [20] found significant age-based variations in the PACIC scores in the primary care environment. Younger individuals showed higher PACIC scores, according to Cramm and Nieboer [21]. The fact that younger people are more likely to report high PACIC ratings may reflect variations in doctors’ attitudes toward various patient populations. Nonetheless, the information is helpful since it implies that a key component of implementing CCM is making sure that all patient groups receive the same level of benefit from improvements in chronic illness care. This study’s finding corroborates those of several studies [22,23], wherein the authors denoted that there was no relationship between the PACIC scores and the number of years since a disease diagnosis. Another study showed that women’s PACIC scores were lower than those of men [24], which contradicts the current study’s findings. Gender differences still existed even after accounting for factors including age, educational attainment, marital status, occupation, housing situation, physical activity, and some chronic diseases such as diabetes and obesity [24]. This showed that the gender difference in the PACIC scores was not explained entirely by sociodemographic profiles and chronic illness conditions. An earlier finding [21] that showed no significant correlation between education and the PACIC scores additionally supports this finding. However, it contradicts the previous finding that demonstrated a link between education and PACIC scores [19]. In perspective, education is anticipated to improve one’s ability to control oneself, obtain essential care, and comply with rules [19]. As a result, the researchers anticipate that, over time, there will be noticeable correlations between schooling and PACIC scores. Conversely, civil status was found to have a strong correlation to the PACIC, which suggests that living with someone is linked to a greater impression of social support, which influences the PACIC positively. The living circumstances and social support have a correlation that is consistent with earlier research that showed a link between living alone and having little social support [25]. The process by which this structure may have an impact involves social support, which is regarded as a structural aspect of social connections [26]. Previous researchers made similar findings; they discovered that individuals with cardiovascular diseases benefited most from having a companion who gave them high-quality support [27]. The PACIC can be used as a monitoring tool to evaluate the effectiveness of the delivery of chronic care. These study results contribute to the significance of understanding that patient evaluations of chronic care delivery may be influenced by patient characteristics, which is in addition to the treatment that the patients obtained.

No significant relationship was found between age, civil status, years of being diagnosed with the disease, educational attainment or occupation, and GSE. This implies that, regardless of age, civil status, years of being diagnosed with the disease, or educational attainment, the GSE does not change. In contrast, sex was found to have a strong correlation with the GSE, which implies that, whether the respondents are male or female, there is variation in the GSE. Previous studies revealed that there were no statistically significant links between GSE and sex [28,29]. This result, on the other hand, contradicts the findings of other studies [30,31,32,33], in which the authors concluded that the older the patient when the disease first manifested, the lower was their cardiac self-efficacy. This is because as patients get older, they have less energy and willpower to take care of themselves, and the sickness becomes worse. The study finding also states that years of being diagnosed with the disease are not associated with the GSE. This validates the finding of Salari and colleagues [30]. However, there are studies that contradict this finding. For example, in the study conducted by Bos-Touwen and colleagues, the patients with longer disease duration had higher self-efficacy because they learned to take better care of themselves over time [34]. The heterogeneous demographic of the study may be the reason for the inconsistent outcomes. The results of this study also differ from those of a study by Davis et al. [35]. that found that self-management practices were influenced by civil status both directly and indirectly through self-efficacy. In addition, higher social support increases the likelihood that healthy actions and coping mechanisms will be positively reinforced, which in turn increases a person’s self-efficacy in managing his or her illness [36]. Similarly, in a recent study, researchers [37] found that people with more support had better self-efficacy, while people with less instrumental support had a greater improvement in self-efficacy over time.

People with cardiovascular diseases would therefore benefit from increased self-efficacy when social support does not limit their autonomy. Indeed, it has been discovered that low self-efficacy in individuals with cardiovascular disease is strongly influenced by education [38]. Low educational achievement may therefore be linked in this study to low self-efficacy. Improved cardiac self-efficacy was correlated with improved cardiac knowledge, which is consistent with prior findings [39]. These findings are helpful for patient-centred health therapies, which should look into both psychological and physiological components. For the health of patients with cardiovascular diseases, self-efficacy is at least as important as cardiac function, and it may be modified more easily. Several disease management programs have been shown to boost participant self-efficacy by having them perform the desired behaviour successfully [40]. Peer leadership, cognitive symptom management techniques, health communication training, and health-related problem solving are all important components of this and other successful disease management programs [41]. Taken together, these findings indicate that more research is needed to determine whether improving self-efficacy mediates behaviour change in chronic disease patients.

### 4.2. Relationship between PACIC and GSE

In the present study, the participants perceived that they had occasionally received care; however, they still perceived themselves to have better self-efficacy. This suggests that self-efficacy continues to increase as people age and gain new knowledge, experiences, and perspectives, thus enabling nurses to establish caring behaviour to assist their cardiovascular disease patients in increasing their self-efficacy as well. This indicates that the greater the care and support provided by healthcare providers, the greater is the patients’ self-efficacy in maintaining their own health. As a result, proper counselling by healthcare providers such as doctors and nurses, appropriate referrals to interdisciplinary professionals, involving clients in disease management goal setting, adequate follow-up, and concern by the healthcare provider can improve patients’ ability to self-care significantly [30]. One study supports the notion that productive interactions with healthcare professionals and high chronic care quality are linked to improved patient self-management abilities [21]. These findings are illuminating when considering the significance of self-efficacy in leading a healthy lifestyle and managing cardiovascular disease [30]. Further, paying attention to patients, interacting with them physically in a way that respects their culture, offering encouragement and empathetic support, and being friendly and welcoming could all enhance patients’ ability to master their own level of self-efficacy [42]. The information offered here might help healthcare professionals work more efficiently in clinical settings. The in-service training programmes offered to healthcare professionals may benefit from these findings as well. Such a finding suggests directions for additional study into the relationship between patient self-efficacy and the frequency of healthcare provider behaviour.

In this study, patient activation had a moderate positive correlation with GSE which means that if patients’ activation improves, GSE will also improve. Indeed, patients are driven to manage their own healthcare because they believe they have a critical role to play in the process and are confident in their ability to do so [43]. Patient activation implicates the capacity for independent management of healthcare [44]. The current result holds true to the earlier study of Mirmazhari and colleagues [45], where they found an important beneficial association between patient activation and self-efficacy. In context, patients may become more active in their care management for this reason by participating in treatment decision-making and establishing shared decision-making [45]. Nurses and physicians can better understand patients by knowing their level of activation vis–vis with the GSE. This will allow them to develop more individualized treatment plans that will better fulfil patients’ requirements and better utilize available resources. Meanwhile, goal setting had a moderate positive correlation with GSE which means that when patients set greater objectives for themselves, they are more likely to attain them, and are more firmly committed when their perceived self-efficacy is improved. This present finding confirms earlier findings that those who have better self-efficacy tend to set higher goals [46].

Finally, there was a strong correlation between the delivery system and GSE which suggests that the provision of respectful care and appropriate communication particularly for cardiovascular patients, appears to be an essential initial step in fostering self-efficacy. As stated in two other studies, the authors discovered that patients with heart disease had a high level of capability and self-efficacy [47,48]. According to one study, the PACIC was linked to better patient-centred outcomes and self-management practices in an adult population with chronic diseases, such as cardiovascular disorders [49]. Two other earlier studies found that patients with cardiovascular disease and hypertension lacked self-efficacy and performed poorly on self-care behaviour [38,50]. Another study discovered that patients’ self-efficacy was moderate after coronary artery bypass graft [51]. The disparities in these findings could be attributed to cultural, perception, and belief differences among the study populations [38]. This influences nurses seeking effective strategies to increase patients’ self-efficacy, particularly through inclusive approaches such as innovative technologies.

### 4.3. Implications for Self-Management Programs

This study contributes to self-management programs for patients with CVD. In perspective, effective self-management includes the capacity to keep track of one’s health and to develop the understanding, behavioural, and emotional reactions required to uphold an acceptable standard of living. In the realm of health and social care, the concept of self-management is well known which according to Al-Maskari et al. [52]. is an active process led by the patient that entails particular tasks in order to accomplish disease management objectives. In the context of this study, PACIC and GSE can be involved in self-management programs for patients with CVD. Such a program needs to be associated with methods of overcoming obstacles through self-help, self-reliance, and family and community support. Moreover, this study implicates adherence to understanding the behaviour of the patients. Giving patients quick feedback on the effects of their behaviour is one way to improve patient self-regulation [53]. For a number of health outcomes, including body weight, blood pressure, and physical activity, self-monitoring of health markers and behaviour outside of the professional context has been utilized as an effective technique to evaluate treatment response and promote adherence [53] relating to GSE. Moreover, this study contributes to hospitals, national quality monitoring agencies, and other stakeholders interested in identifying ‘patient-centred’ quality of care metrics who will find this information helpful. In addition, this study contributes to health systems and other stakeholders in evaluating and enhancing the standard of care for patients with chronic diseases, particularly those with cardiovascular conditions. Although, we do advise using caution when implementing these findings in a clinical setting. Consequently, additional research examining this link over a longer time frame is advised so that patients have sufficient time to increase their self-efficacy. The strength of this study lies in the use of a validated tool in the Arabic translation, where patients easily understood the context and content of the questions.

#### Study Limitations

The authors noted convenience sampling as a study limitation that might reduce the generalizability of the findings; hence, careful consideration must be given to how the data are interpreted. Moreover, during the data collection, we supervised the participants while answering the questionnaire and this could lead to bias which could select answers according to the supervisor’s expectations. Also, we failed to include the patient’s characteristics, and scores on PACIC and GSE based on their characteristics would be beneficial. Furthermore, one scale (e.g., PACIC) could be more applicable to a certain condition (e.g., acute decompensated heart failure). Finally, the receiver operating characteristic (ROC) curve correlation representation for each parameter was not provided; this can be addressed in further investigations since it will provide greater clarity for the research parameters mentioned.

## 5. Conclusions

Based on the findings of the study, it is possible to conclude that certain modifiable factors can improve the self-efficacy of patients with cardiovascular disorders. Proper support, guidance, and counselling from healthcare providers can all significantly help to improve patients’ self-efficacy. A strong relationship was found between the delivery system and the GSE. Such a result illuminates the value of self-efficacy and patient involvement as self-management techniques for cardiovascular illnesses. Future cardiovascular illness self-management initiatives should concentrate on enhancing patient self-efficacy by adopting the PACIC. More studies should be carried out on the subject of the best methods for illness management interventions and the significance of patient self-efficacy in self-management programs.

## Figures and Tables

**Table 1:** Demographic characteristics of the participants. N = 104.

Age	Frequency	Percent
Under 30 years old	39	37.5
Over 30 years old	65	62.5
Civil Status		
Single	35	33.7
Married	69	66.3
Number of Years diagnosed with the disease		
Less than a year	27	26.0
1–2 years	25	24.0
3 years and above	52	50.0
Sex		
Male	43	41.3
Female	61	58.7
Educational Attainment		
High School Level	54	51.9
College Level	34	32.7
Postgraduate Level	16	15.4
Occupation status		
Unemployed	13	12.5
Employed	59	56.7
Retired	32	

**Table 2:** Correlations between demographics, GSE, and PACIC.

	Age	Civil Status	Years of Having the Disease	Sex	Educational Attainment	Occupation	PACIC	GSE
Age	1							
Civil Status	0.121	1						
Years having the disease	0.293 **	0.326 **	1					
Sex	−0.287 **	−0.061	0.008	1				
Educational Attainment	−0.250 *	0.117	−0.014	0.194 *	1			
Occupation	−0.277 **	−0.002	−0.240 *	0.009	0.083	1		
PACIC	0.062	−0.867 **	−0.174	0.180	0.125	0.206 *	1	
GSE	0.070	0.013	0.095	0.795 **	0.088	0.115	0.881 **	1

** Correlation is significant at the 0.01 level (2-tailed). * Correlation is significant at the 0.05 level (2-tailed).

**Table 3:** Perceived level and relationship of PACIC and GSE.

	Mean	Std. Deviation	GSE	Patient Activation	Delivery System	Goal Setting	Problem Solving	Follow Up
GSE	25.28	4.945	1	0.397 **	0.507 **	0.360 **	0.228 *	0.278 **
Patient Activation	2.71	1.150	0.390 **	1	0.777 **	0.266 **	0.241 *	0.210 *
Delivery System	3.07	1.203	0.507 **	0.777 **	1	0.380 **	0.339 **	0.340 **
Goalsetting	2.58	0.761	0.360 **	0.266 **	0.380 **	1	0.503 **	0.703 **
Problem Solving	2.78	1.238	0.228 *	0.241 *	0.339 **	0.503 **	1	0.563 **
Follow-up	2.59	0.919	0.278 **	0.210 *	0.340 **	0.703 **	0.563 **	1
Total Mean	2.75	1.053						

** Correlation is significant at the 0.01 level (2-tailed). * Correlation is significant at the 0.05 level (2-tailed).

## Data Availability

The data presented in this study are available on request from the corresponding author.
